# Tai Chi Ameliorates Coronary Heart Disease by Affecting Serum Levels of miR-24 and miR-155

**DOI:** 10.3389/fphys.2019.00587

**Published:** 2019-05-29

**Authors:** Yang Li, Haiyang Zhang, Yushi Wang

**Affiliations:** Department of Cardiovascular Center, The First Bethune Hospital of Jilin University, Changchun, China

**Keywords:** Tai Chi, self-care ability, coronary heart disease, cardiac function, serum microRNA

## Abstract

The protective role of Tai Chi in coronary heart disease (CHD) has been widely reported. However, the exact molecular mechanism remains unclear. Serum levels of miR-24 and miR-155 have been found to potentially be involved with CHD risk. Thus, the effects of Tai Chi on CHD risk were explored by measuring serum levels of miR-24 and miR-155. A total of 326 CHD patients were evenly divided into the Tai Chi (TG) and control (CG) groups. The activities of daily living ability (ADL) and exercise of self-care agency (ESCA) scores were compared between the two groups. Left ventricular ejection fraction (LVEF), SF-36 life quality, self-rating anxiety scale (SAS) and self-rating depression scale (SDS) were used to evaluate subjects’ cardiac function, quality of life, anxiety, and depression. Serum levels of miR-24 and miR-155 were measured by a real-time quantitative polymerase chain reaction (RT-qPCR). After a 6-month Tai Chi intervention, the ESCA, ADL, LVEF, and SF-36 scores in the TG group were higher than those in the CG group (*p* < 0.05). The time of arrhythmia and atrioventricular block recovery and hospital stay, and the scores of SAS and SDS in the TG group were lower than in the CG group (*p* < 0.05). Serum levels of miR-24 and miR-155 in the TG group were also lower than in the CG group (*p* < 0.05). In addition, serum levels of miR-24 and miR-155 were negatively associated with the ESCA, ADL, LVEF and SF-36 scores, and had adverse effects on life quality. Altogether, these present findings demonstrate that Tai Chi improves CHD prognosis, by affecting serum levels of the miR-24 and miR-155.

## Introduction

Cardiovascular disease is the leading cause of death and disability worldwide (Santulli, 2013). The number of coronary heart disease (CHD) patients continues to increase year on year (Fukumoto et al., 2017) and reducing the mortality rate has become an urgent social concern (Virtanen et al., 2017). Tai Chi is an ancient practice used to improve cardiovascular health. It improves aerobic endurance and psychosocial well-being and may be a promising exercise option in cardiac rehabilitation. With the improvement of nursing practices, Tai Chi has become a standard method for CHD care (Salmoirago-Blotcher et al., 2017; Liu et al., 2018). Tai Chi is a comprehensive and long-term program designed to reduce the physical and psychological impact of heart disease on patients, and improves their social psychology (Blake and Hawley, 2012; Zheng et al., 2014, 2015).

However, the molecular mechanism employed in the protective role of Tai Chi on CHD progression, remains widely unknown. miRNAs are short non-coding RNAs that affect gene expression by binding to the 3′-UTR of target mRNAs and inhibit protein production via the destabilization of mRNA and translational silence. They can also target promoter regions and affect gene transcription (Cannell et al., 2008). microRNAs and their target genes may be involved in heart disease (Lu et al., 2018). Serum levels of miR-24 have been found to be associated with heart failure mortality (Zhang et al., 2018). Serum miR-155 was reported as a biomarker for CHD diagnosis (Jia et al., 2017). SCN5A gene encodes voltage-gated Na^+^ channel NaV1.5, which depolarizes cardiac action potential. Mutant SCN5A is involved in heart failure-related sudden cardiac death, and miR-24 suppresses SCN5A expression (Zhang et al., 2018). Angiotensin receptor type 1 (AT1R) polymorphism has been found to be associated with CHD risk (Buraczyńska et al., 2003; Watkins et al., 2006), and miR-155 regulates AT1R expression (Blanco et al., 2012). Exercise can reduce the expression of miR-155 in an animal model (Rocha et al., 2018), but no report for miR-24 has been published yet. Both miRNAs regulate the transcripts of the brain-derived neurotrophic factor (BDNF) (Zhao and Srivastava, 2007; Xu et al., 2017), which is related to exercise effects in the human body as well as depression (Lu et al., 2014).

Serum miR-24 and miR-155 can be a potential target for CHD therapy. Tai Chi may improve CHD symptoms by affecting serum levels of miR-24 and miR-155. Therefore, we explored the effects of Tai Chi on CHD patients, by analyzing serum levels of miR-24 and miR-155.

## Materials and Methods

### Participants

This study was approved by the Ethics Committee of The First Hospital of Jilin University Bethune (Changchun, China) and written informed consent was obtained from each participant before data collection. All participants were diagnosed with CHD. From March 2014 to March 2016, 1788 patients were admitted to the critical care unit of our hospital’s cardiology department.

### Inclusion Criteria

The patients were diagnosed with CHD according to the New York Heart Association (NYHA) standards (Hu et al., 2017). The patients had the following demographic characteristics: angiographically proven CHD, heart function between I and III levels, LVEF <40%, and older than 18 years without dementia and an acute coronary event within the last 3 months. The spouse or family of patients served as caregivers, and they accompanied the patients at the time of enrollment.

### Exclusion Criteria

The exclusion criteria were as follows: unclear patient awareness; the occurrence of shock and need for defibrillation to restore cardiac reflex; pregnant or lactating females; the CHD risk associated with changes in systemic inflammation, such as rheumatoid arthritis and immunological deficiency; receiving medications including systemic steroids, non-steroidal drugs, and hormone therapy and failing to comply with follow-up visits.

### Patients Grouping

**FIGURE 1:**
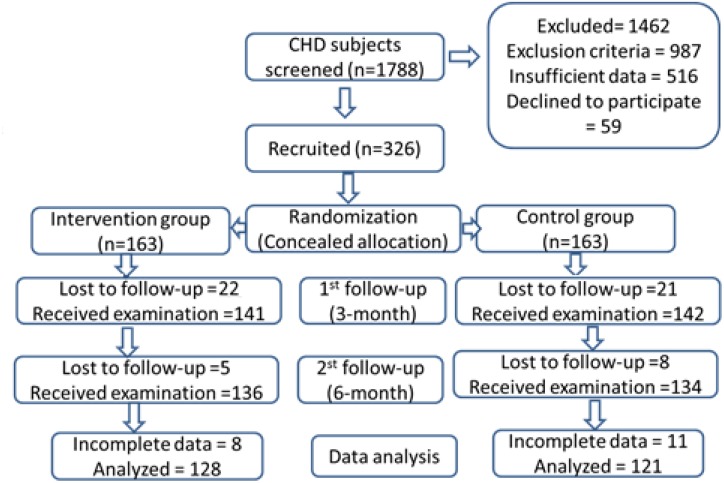
Study flow diagram.

A total of 326 patients met the inclusion criteria and were enrolled in this study. Using a random number table, the subjects were evenly and randomly divided into the Tai Chi group (TG) and control group (CG). After a 6-month follow-up, 128 and 121 patients in TG and CG groups completed the present study, respectively (Figure 1).

### Tai Chi Intervention

All patients were given conventional treatment and care, including the treatment of CHD symptoms, appropriate diets, exercises, medicine, and psychological therapy. The Tai Chi program consists of five movements and 24 forms in a Yang style (Verhagen et al., 2004). Patients in the TG group were trained by Tai Chi tutors. The classes lasted for 1 h per day, and the whole program was performed in the same place. In the CG group, the patients received physical exercise for 1 h per day according to previous reports (Aliabad et al., 2014).

### Exercise of Self-Care Ability Test (ESCA)

Self-care ability was measured using a questionnaire provided by an instrument to measure ESCA according to a previous report (Kearney and Fleischer, 1979). The questionnaire includes four aspects: self-care skills (12 items), self-responsibility (eight items), self-concept (nine items), and health knowledge (14 items). The ESCA scores were divided into high (116–172), medium (58–115), and low (0–57) levels.

### Assessment of Daily Living Ability (ADL)

The ADLs were measured according to a previous report (Xiaolan, 2017). The scale was divided into two parts with 14 indicators, which were scored from 1 to 4. The scale was scored as follows: 1, daily living activity could be performed; 2, there were some difficulties when daily activities were performed; 3, help was needed when daily activities were performed and 4, daily activities could not be performed. Individuals who scored 1 were considered normal, and those who scored 4 to 6 were considered to have a decline in function or functional dependence. The scale score ranged from 1 to 56. Scores ≤20 indicated that the patients could take care of themselves, and scores >20 indicated that the patients could not take care of themselves. The homogeneity reliability was 0.84 and the CVI was 0.93.

### Measurement of Cardiac Function

LVEF was measured to evaluate the improvement of cardiac function. Transthoracic echocardiography was performed in all patients using Vivid E9 (GE Healthcare, Milwaukee, WI, United States). LVEF was calculated from the apical four- and two-chamber views using a Simpson’s biplane method (Duncan et al., 2011). We investigated the time of arrhythmia and atrioventricular block recovery, and the hospitalization duration after nursing care.

### Measurement of Life Quality

Life quality was measured according to eight aspects of SF-36 life quality: physiologic functioning (PF), role physical (RP), bodily pains (BP), general health (GH), vitality (VT), social functioning (SF), role limitation due to emotional problems (RE), and mental health (MH). These items were scored from 0 to 100. The scores indicated the life quality.

### Anxiety and Depression Measurement

The self-rating anxiety scale (SAS) and self-rating depression scale (SDS) are simple tools used to assess depression (Taylor et al., 2005) and emotional disturbance. Depression (Jiang et al., 2018) and poor emotional control (Potijk et al., 2016) are considered significant predictive risk factors for CHD patients. Moreover, depression may affect the recovery of CHD patients (Shen and Gau, 2017). A meta-analysis showed that exercise of continuous moderate-intensity significantly increases LVEF in patients with heart failure (Tucker et al., 2018). Exercise can reduce depressive and anxiety and improve outcomes of heart failure patients (Chiala et al., 2018).

The physical and psychological status (anxiety and depression) of patients in the CG and TG groups was investigated using SAS (Li et al., 2016) and SDS (Sahni and Agius, 2017) between CG and TG groups. The following criteria were considered using the scores: below 50, without depression/dysphoria; 50–59, mild depression/dysphoria; 60–69, moderate depression/dysphoria and above 70, with high depression/dysphoria.

### Measurement of Serum Levels of miR-24 and miR-155

Five milliliters of blood was obtained from each subject via venipuncture, and the serum was isolated from the blood using centrifugation at 2000 × *g* for 10 min. Serum total RNA was extracted using a kit (Ambition, Life Technologies, Carlsbad, CA, United States) according to the manufacturer’s instructions. Serum levels of miR-24 (forward primer: 5′-GTCGTATCCAGTGCAGGGTCCGAGGTATTCGCACTG-3′; reverse primer, 5′-GTGCAGGGTCCGAGGT-3′); and miR-155 (forward primer: 5′-GTCGTATCCAGTGCAGGGTCCGAGG-3′, reverse primer: 5′-TCGCACTGGATACGACCCCCTA-3′) were measured by using real-time quantitative polymerase chain reaction (RT-qPCR). U6 was used as a control using snRNA-forward primer, CTCGCTTCGGCAGCACA, and snRNA-reverse primer, AACGCTTCACGAATTTGCGT.

### Statistical Analysis

All data were presented as mean values ± SD (standard deviations). A multivariate analysis of variance was used to assess the conditional effect of each variable (Hawkes et al., 2016a,b). It also reduced the number of statistical tests run on the same set of variables, thus controlling for alpha slippage. The statistical difference for the numbers (gender, smoking, and marital status) between the two groups was measured using the χ2 test. Other variables were analyzed using a multivariate analysis with adjusted hazard ratios and 95% confidence intervals. An association test was used to test the coefficients of two variants. All data were analyzed using SPSS 20.0 (SPSS Inc., United States). *p* < 0.05 was considered statistically significant.

## Results

### Basic Clinical Characteristics

Demographic variables included education, occupation, age, gender, BMI, monthly income, residence, NYHA class, and marital status. The average age of patients in the TG and CG groups were 62.6 ± 5.62 and 64.4 ± 5.29 years, respectively. Educational levels were similar in both groups. In addition, all patients in the two groups had a similar NYHA functional class, and no class IV was found. Clinical characteristics showed that a statistical difference was insignificant in the two groups (Table 1, *p* > 0.05).

**Table 1:** Clinical and demographic characteristics between two groups.

	Tai Chi group (*n* = 163)	Control group (*n* = 163)	*f*-ratio	*p* value
Age (years)	63.61 ± 6.62	65.44 ± 5.79	0.101	0.759
Gender, male (%)	72 (44.2)	68 (41.7)	χ^2^ = 0.200	0.654
BMI, weight/height^2^ (kg/m^2^)	28.9 ± 3.5	29.4 ± 3.7	1.882	0.207
Smoking, cases (%)	26 (16)	28 (17.2)	0.089
Marital status, married (%)	145 (89.0)	149 (91.4)	0.000	1.000
Monthly income, RMB	4982 ± 3256	4618 ± 3029	2.456	0.127
Region	Changchun city	Changchun city		
Occupation, cases (%)			0.000	1.000
Unskilled, cases (%)	25 (15.3)	27 (16.6)		
Professional, cases (%)	78 (47.9)	75 (46)		
Skilled, cases (%)	60 (36.8)	61 (37.4)		
Education			0.000	1.000
Elementary, cases (%)	36 (22.1)	34 (20.9)		
Middle school, cases (%)	30 (18.4)	32 (19.6)		
Professional school, cases (%)	24 (14.7)	22 (13.5)		
High school, cases (%)	43 (26.4)	42 (25.8)		
University degree, cases (%)	30 (18.4)	33 (20.2)		
NYHA class			0.000	0.996
I, cases (%)	36 (22.1)	38 (23.3)		
II, cases (%)	49 (30.1)	58 (35.6)		
III, cases (%)	77 (47.2)	67 (41.1)		
IV, cases (%)	0	0		

*Data are reported as means ± SD or number and percentages. BMI, body mass index; CHF, chronic heart failure; LVEF, left ventricular ejection fraction measurement; NYHA, New York Heart Association. The statistical test was performed with a chi-square test and multivariate analysis.*

### Tai Chi Increased the ESCA and ADL Scores of CHD Patients

After a 6-month follow-up, the ESCA and ADL scores in the TG group were 122.5 ± 13.4 and 45.7 ± 6.5, respectively, which were better than that in the CG group (105.4 ± 12.5 and 39.6 ± 4.8, respectively) (Table 2, *p* < 0.05).

**Table 2:** Comparison of ESCA score and ADL score before and after nursing care between two groups.

Groups	ESCA score			ADL score		
	0 month	3 months	6 months	0 month	3 months	6 months
Tai Chi group	86.6 ± 11.7	96.2 ± 12.6	122.5 ± 13.4	33. 3 ± 8.3	38.2 ± 8.9	45.7 ± 6.5
Control group	87.3 ± 10.7	90.3 ± 11.8	105.4 ± 12.5	32.0 ± 7.6	34.1 ± 6.5	39.6 ± 4.8
*f*-ratio	0.146	0.458	2.964	0.247	2.53	2.76
*p*	0.412	0.359	0.00	0.352	0.038	0.007

*n = 128 in Tai Chi group and n = 121 in control group. The statistical test was performed with a multivariate analysis.*

### Tai Chi Improved the Symptoms of CHD Patients

The recovery time of arrhythmia in the TG group was shorter than in the CG group (Table 3, *p* < 0.05). LVEF was 47.3% ± 3.4% in the TG group, which was higher than that in the CG group (42.6% ± 3.1%, Table 3, *p* < 0.05); However, the time of atrioventricular block recovery and hospitalization duration in the TG group was lower than that in the CG group (*P* < 0.05). Tai Chi improved the clinical symptoms of CHD patients.

**Table 3:** Comparison of symptom improvement of CHD patients between two groups.

Groups	Recovery time of arrhythmia (days)	LVEF%	Recovery time of atrioventricular block (days)	Hospital stay (days)
Tai Chi group	4.5 ± 1.2	47.3 ± 3.4	3.3 ± 1.6	16.4 ± 2.5
Control group	6.3 ± 1.3	42.6 ± 3.1	5.5 ± 1.4	20.5 ± 4.8
*f*-ratio	2.785	1.953	3.652	2.218
*p*	0.015	0.043	0.006	0.032

*n = 128 in Tai Chi group and n = 121 in control group. The statistical test was performed with a multivariate analysis.*

### Tai Chi Improved the SF-36 Life Quality Scores After Treatment

Changes in the life quality scores were measured using SF-36 scores before and after Tai Chi intervention. The average SF-36 scores in the TG and CG groups were 61.5 ± 7.4 and 44.0 ± 5.3 after a 6-month follow-up, respectively. SF-36 life scores showed that the life quality of the TG group was improved in eight aspects, including PF, RP, BP, GH, VT, SF, RE, and MH. Comparatively, the patients in the CG group showed improvement in four aspects, including PF, RE, SF, and BP (Table 4).

**Table 4:** Comparison of the SF-36 life quality (a score between 0 and 100) between two groups.

	Tai Chi group, *n* = 128	Control group, *n* = 121	*p* values
**0 month**			
PF	33.9 ± 6.9	35.1 ± 4. 3	0.712
RP	32.7 ± 5.3	34.4 ± 6. 2	0.342
RE	26.2 ± 6.3	27.9 ± 5.6	0.885
SF	42.5 ± 4.1	41.5 ± 3.1	0.873
GH	56.9 ± 5.8	54.5 ± 4.3	0.801
BP	37.6 ± 6.7	37.9 ± 3.6	0.718
VT	35.9 ± 3.7	38.1 ± 4.1	0.523
MH	45.4 ± 5.3	43.3 ± 5.1	0.429
Average	39.5 ± 5.8	36. 8 ± 4.9	0.278
**3-month follow-up**			
PF	45.7 ± 7.4	38.9 ± 5.8	0.023
RP	42.8 ± 4.0	35.6 ± 5.1	0.018
RE	32.7 ± 4.6	28.1 ± 6.5	0.032
SF	53.7 ± 6.3	40.1 ± 5.2	0.009
GH	51.4 ± 5.8	47.3 ± 3.5	0.068
BP	39.8 ± 6.5	38.1 ± 4.0	0.187
VT	41.5 ± 7.3	37.2 ± 5.5	0.094
MH	46.0 ± 8.8	44.3 ± 6.7	0.292
Average	44.6 ± 6.7^∗^	39.1 ± 4.3^∗^	0.045
**6-month follow-up**			
PF	62.1 ± 10.8	42.9 ± 6.3	0.001
RP	65.8 ± 3.9	36.7 ± 4.1	0.001
RE	61.2 ± 5.7	44.9 ± 7.3	0.001
SF	63.4 ± 7.4	33.8 ± 5.6	0.001
GH	62.1 ± 6.3	56.1 ± 3.7	0.014
BP	54.8 ± 7.2	48.8 ± 4.5	0.001
VT	66.7 ± 8.5	36.3 ± 6.2	0.001
MH	48.1 ± 9.6	42.1 ± 7.3	0.042
Average	61.5 ± 7.4^∗^	40.0 ± 5.3^∗^	0.001

*Follow-up was 3 months. SF-36 life quality includes PF, physiologic functioning; RP, role physical; BP, bodily pains; GH, general health; VT, vitality; SF, social functioning; RE, role of limitation due to emotional problems; and MH, mental health. The statistical test was performed with a multivariate analysis.*

### Tai Chi Improved Depression Symptoms After Treatment

Before Tai Chi intervention, the statistical difference was insignificant between the two groups (*p* > 0.05, Table 5). Compared with those in the CG group, the average values of SAS and SDS decreased significantly in the TG group, and the difference was statistically significant after a 6-month follow-up (Table 5, *p* < 0.05). All these results demonstrated that Tai Chi improved depression after treatment.

**Table 5:** Comparison of SAS/SDS scores between groups.

Groups	SAS			SDS		
	0 month	3 months	6 months	0 month	3 months	6 months
TG	55.6 ± 4.6	50.1 ± 4.8	32.3 ± 4.5	57.3 ± 10.5	48.3 ± 9.9	39.2 ± 9.3
CG	53.9 ± 6.9	53.7 ± 6.1	43.9 ± 5.6	56.5 ± 6.9	54.1 ± 7.0	53.6 ± 8.7
*f*-ratio	0.263	0.398	3.598	0.156	1.826	3.247
*P*	0.512	0.256	0.001	0.672	0.039	0.003

*SAS, self-rating anxiety scale; SDS, self-rating depression scale. The statistical test was performed with a multivariate analysis.*

### Tai Chi Reduced the Serum Levels of miR-24 and miR-155

**FIGURE 2:**
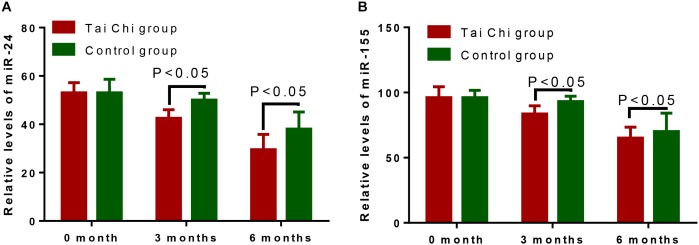
The effects of Tai Chi on serum levels of miR-24 and miR-155. **(A)** the effects of Tai Chi on serum levels of miR-24. **(B)** the effects of Tai Chi on serum levels of miR-155.

Before Tai Chi intervention, the statistical difference for serum miR-24 levels was insignificant (Figure 2A, *p* > 0.05). After the 3- and 6-month training, Tai Chi reduced the serum levels of miR-24 (Figure 2A, *p* < 0.05). Similarly, before Tai Chi intervention, the statistical difference for serum miR-155 levels was insignificant (Figure 2B, *p* > 0.05). After the 3- and 6-month intervention, Tai Chi reduced the serum levels of miR-155 (Figure 2B, *p* > 0.05).

### Serum Levels of miR-24 and miR-155 Had Adverse Effects on Health-Related Quality of Life in CHD Patients

**FIGURE 3:**
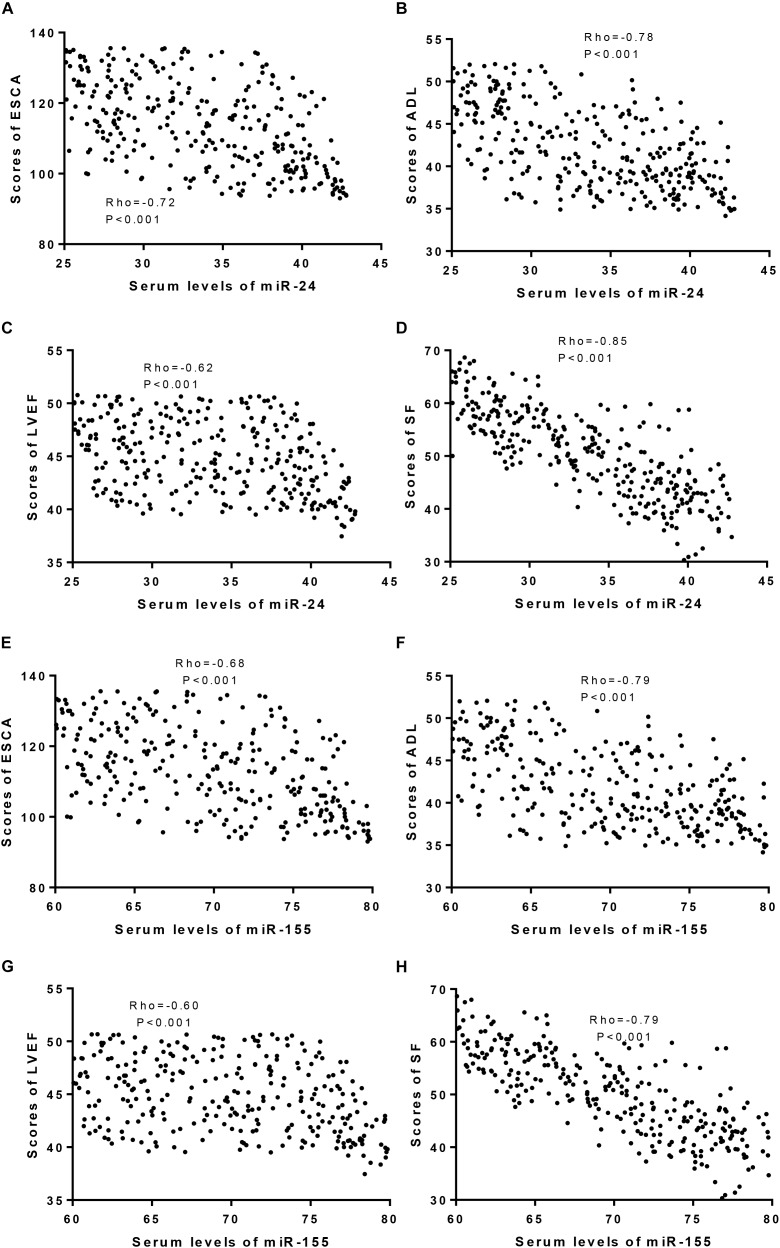
An association test was used to test the coefficients between serum levels of miroRNA and scores of ESCA, ADL, LVEF and SF-36. **(A)** The relationship between serum levels of miR-24 and scores of ESCA. **(B)** The relationship between serum levels of miR-24 and scores of ADL. **(C)** The relationship between serum levels of miR-24 and scores of LVEF. **(D)** The relationship between serum levels of miR-24 and scores of SF-36. **(E)** The relationship between serum levels of miR-155 and scores of ESCA. **(F)** The relationship between serum levels of miR-155 and scores of ADL. **(G)** The relationship between serum levels of miR-155 and scores of LVEF. **(H)** The relationship between serum levels of miR-155 and scores of SF-36. There is a strong negative relationship if rho <−0.5.

An association test showed that serum miR-24 levels had a negative relationship with ESCA (Figure 3A), ADL (Figure 3B), LVEF (Figure 3C), and SF-36 (Figure 3D). Similarly, serum miR-155 also had a negative relationship with the ESCA (Figure 3E), ADL (Figure 3F), LVEF (Figure 3G), and SF-36 scores (Figure 3H). The results suggested that serum miR-24 and miR-155 levels had adverse effects on the quality of life in CHD patients.

## Discussion

Traditional Chinese exercises are very popular in China, especially Tai Chi, which is of great benefit to people’s health and heart-related diseases. Tai Chi is suitable for any population as well as different ages. Tai Chi intervention is low risk and can be used to improve the quality of life for cardiovascular patients, especially CHD patients (Liu et al., 2018). The initial concept of Tai Chi is to make full use of available human resources and to motivate patients’ initiative and to reduce the intensity of nurses’ work. Depression and anxiety are well-known indicators of poor outcomes in CHD patients (Palacios et al., 2018). CHD patients undergoing percutaneous coronary intervention have a higher rate of depression than the general population. Treating depression has proven to be effective in improving outcomes of CHD patients (Feng et al., 2017).

In this study, anti-anxiety (Zheng et al., 2018) and anti-depressant (Rawtaer et al., 2015; Siu et al., 2017) exercises of Tai Chi were applied to observe their impact on a patients self-care ability, ADLs, and disease prognosis. The results showed that ESCA, ADL and SF-36 scores in the TG group were significantly improved, and better than in the CG group (Table 2, *P* < 0.05). Tai Chi intervention reduced the time of arrhythmia recovery and hospitalization duration. These results are consistent with previous reports. A Tai Chi exercise regimen can improve the physical function and ability of frail elderly individuals and reduce the demand for long-term care (Nomura et al., 2011). Tai Chi has been reported to ameliorate subsyndromal depression and anxiety in the elderly population (Rawtaer et al., 2015). Tai Chi is an effective strategy to enhance cognitive function and maintain movement abilities in patients with instrumental ADLs and mild cognitive impairment (Siu and Lee, 2018). Moreover, a previous study found that a 12-week Tai Chi intervention improved the LVEF in the patients with chronic kidney and cardiovascular diseases (Shi et al., 2014). Tai Chi has provided novel ideas and directions for the management of atrial fibrillation (the most common cardiac arrhythmia) management.

In the TG group, cardiac function was improved, and symptom recovery time was shortened (*P* < 0.05). The results demonstrated the value of Tai Chi in heart disease care. After intervention, patients became familiar with heart disease-related knowledge, strengthened their self-care skills, and enhanced their daily activities so as to improve their cardiac function and prognosis.

Serum miR-24 and miR-155 levels are associated with the risk of heart failure (Esmaeili-Bandboni et al., 2018; Zhang et al., 2018). The present work showed that Tai Chi intervention reduced the serum levels of miR-24 and miR-155 in CHD patients (Figure 2, *p* < 0.05). Furthermore, serum levels of miR-24 and miR-155 were negatively related with ESCA, ADL, LVEF and SF-36 scores (Figure 3, *p* < 0.05). Tai Chi intervention improved CHD symptoms by reducing serum levels of miR-24 and miR-155.

Tai Chi improved the self-care and living ability by increasing ESCA, ADL and SF-36 scores. It strengthened cardiac function by increasing the percentage of LVEF. In addition, Tai Chi reduced SAS and SDS scores significantly, and should be developed as a potential method in the prevention of CHD. Anxiety and depression are considered to have genetic roots (Petito et al., 2016), thus, Tai Chi may improve SAS and SDS scores by affecting the genes related to anxiety and depression.

Despite our findings, the present work has some limitations. First, Tai Chi could not be provided for most CHD patients because of the lack of well-trained Tai Chi tutors. Exploring the therapeutic effect of Tai Chi on CHD is important; Second, a molecular link between Tai Chi and prevention of CHD progression, will provide insights into the functional role of Tai Chi in CHD therapy; Third, different psychotherapists will have different skills, which might lead to different therapeutic results. A unified public platform for Tai Chi is highly demanded. Therefore, further work is still needed to address these important issues.

## Conclusion

Tai Chi increased ESCA and ADL, LVEF and SF-36 scores, and significantly reduced SAS and SDS scores. Tai Chi improved the self-care, ADLs, cardiac function, and prognosis of CHD patients. Given these findings, Tai Chi should be developed as a potential method for CHD therapy.

## Ethics Statement

This study was approved by the Ethics Committee of The First Hospital of Jilin University Bethune, and written informed consent was obtained from each adult before data collection. All participants were diagnosed with CHD.

## Author Contributions

HZ and YW designed and performed the experiments. YW analyzed all data. YL wrote the manuscript.

## Conflict of Interest Statement

The authors declare that the research was conducted in the absence of any commercial or financial relationships that could be construed as a potential conflict of interest.
